# Impact of Vaccine and Immunity Passports in the Context of COVID-19: A Time Series Analysis in Overseas France

**DOI:** 10.3390/vaccines10060852

**Published:** 2022-05-26

**Authors:** Samuel d’Almeida

**Affiliations:** Independent Researcher, 75001 Paris, France; samuel.dalmeida@posteo.net

**Keywords:** COVID-19, decision making, human rights/ethics, pandemics, public health/ethics, risk assessment, SARS-CoV-2, social determinants of health, travel, vaccination, viral vaccines

## Abstract

(1) Background: By summer 2021, overseas France turned COVID-19 vaccine and immunity certificates into passports to open travel bubbles. Subsequently, its territories set French records for both COVID-19 and 6-month excess all-cause mortality. (2) Methods: Official time series were collected to compare time correlations between air traffic and COVID-19 transmission and mortality in overseas France, before and after the implementation of immunity passports. (3) Results: Air traffic initially had a reversed relationship with COVID-19, which transitioned into a leader–follower relationship with the introduction of immunity passports. Essentially, air traffic increased 16 days before COVID-19 cases increased (r = 0.61) and 26 days before deaths increased (r = 0.31) in Martinique, 26 days (r = 0.72) and 40 days (r = 0.82) before in Guadeloupe, and 29 days (r = 0.60) and 31 days (r = 0.41) before in Réunion upon introduction of immunity passports. Moreover, air traffic became as correlated as community transmission to COVID-19 mortality in Guadeloupe. (4) Conclusions: Since the introduction of immunity passports, air traffic has been pacesetting COVID-19 within one month for transmission, and within an additional two weeks for mortality in overseas France. Responding to WHO’s call for real-world evidence, this study suggests that COVID-19 passports are not commensurate with health system goals.

## 1. Introduction

Overseas France is a set of French territories distributed worldwide outside the European continent, which remains strongly connected to mainland France [1,2] for familial, tourism, and professional reasons. Most of these territories have reopened twice to non-essential travel—i.e., “travel bubbles”—since the onset of the COVID-19 pandemic: first, in summer 2020, with mandatory testing and quarantine [3], and second, in June 2021, waiving quarantine, and eventually waiving mandatory testing, for travellers with vaccine and immunity certificates, known as “passports” [4].

In June 2021, the Commonwealth of Dominica—a sister island to the French Caribbean—sought to reopen by scrapping quarantine for tourists in possession of a COVID-19 vaccine passport [5]. Subsequently, cruise calls restarted at the capital Roseau [6], and in their wake the shadow of COVID-19 deaths was cast over the island for the first time [7].

Pandemic experts call it a “lemming syndrome”, which is reflected by countries reopening in imitation of their neighbours, rather than by reference to risk-based assessment [8,9].

The Word Health Organization (WHO) forwent plans on vaccine and immunity passports very early [10], describing these as “out of the scope” of the fight against the COVID-19 pandemic [11]. In late 2021, WHO warned against the carte blanche—i.e., the “false senses of security”—packaged with COVID-19 vaccines, which puts at risk both the vaccinated and the unvaccinated, as efficacy proved to wane for preventing mortality and descended into insufficiently preventing transmission [12].

The French Caribbean is notable for being amid the most travelled placed in the world, its trade-type justification being heavily reliant on tourism [13] and being part of the European Union (EU) [14], and yet is far behind the global vaccine schedule [15]. Before COVID-19, each year more than one million air travellers flocked to Guadeloupe and Martinique. This flow, not including cruise passengers, outnumbered islander populations. Visitors were mostly French and other EU citizens—not only tourists, but often relatives [16]. Free movement between Europe and EU regions of overseas France was eventually recovered by the EU’s “green pass” used for travellers [14,17,18].

COVID-19 vaccine and immunity passports were pushed onto the agenda in the light of vaccines’ marketing authorisations by the World Tourism Organization (UNWTO), a sister UN agency of WHO, albeit with distinct constituencies [19]. At that time, UNWTO pushed for seizing the opportunities to revive past travel and tourism economies, which were both deemed collaterals of the pandemic. The terms “safe and seamless travels” and “#SafeTravels” were coined in this context [20,21], calling for the removal of travel obstacles with vaccine and immunity passports—not only travel bans, but the whole package of non-pharmaceutical interventions (NPIs) that were deemed to be hampering travel and tourism, including quarantine regimes, stay-at-home orders, and/or testing requirements [13,22]. This year, UNWTO’s early campaign against “blanket travel restrictions” [23] was disclaimed by WHO, specifying that the discontinuation of COVID-19 border controls should be conditional upon continuous risk-based assessment [24]. In fact, WHO was concerned about the absence of real-world evidence on the aviation sector’s interventions in the context of COVID-19 [25].

Travel bubble schemes between Europe and overseas France started opening on 9 June 2021, following the model of UNWTO’s ethics committee recommendations [26], and those of the European Council [27]. At the time, the French scientific council on COVID-19 (the “Conseil scientifique COVID-19”) was very pessimistic about restarting mass tourism in the overseas territories due to their minimal vaccination coverage and the looming variant of concern, Delta [28].

Shortly afterwards, the French Caribbean topped all levels of all-cause excess mortality and levels of COVID-19 faced by any French territory since the beginning of the pandemic [29], while, on the other side of the globe, French Polynesia reached the highest COVID-19 levels for the WHO Western Pacific region [30,31]. Violent social unrest began in Guadeloupe and Martinique by autumn 2021 [32,33,34], focusing international media attention on the unvaccinated [35,36].

In fact, COVID-19 vaccine acceptance in overseas France was not a foregone conclusion [34]. The immunity passport scheme for travelling to overseas territories was adopted two months ahead of the generalisation of the vaccine mandate for long-distance travel in mainland France (i.e., the “French health pass”) in August 2021 [34,37]. Indeed, the overwhelming majority of overseas France’s residents were not vaccinated upon adoption of immunity passports, unlike those of mainland France [38]. Barely one sixth of the population in the French Caribbean and Mayotte [28], about one fourth in French Polynesia and New Caledonia [39], and one third in Réunion [28] received their first dose of the vaccine. Today, while 60% of the French population have received their first booster vaccines [40] it is only 40% in Réunion [41] and French Polynesia [42], and a little more than 20% in the French Caribbean [43,44].

Ultimately, the unanswered public health calls for engagement in vaccination [15,33,45] created a political impasse [34]. Nevertheless, France and the European Council vowed to keep some “emergency brakes” close to hand with regards to immunity passports [27,28,46], but these were never called into question for overseas France.

## 2. Materials and Methods

### 2.1. Scope, Design and Objectives

This study is observational and based on data made publicly available by official agencies. Its aim is to analyse the impact of vaccine and immunity passports on COVID-19 outcomes, based on time correlations between air traffic in overseas France, and both COVID-19 transmission and mortality.

### 2.2. Inclusion and Data Collection

The study relies on the data published by French central agencies and is limited, by design, only to the inclusion of the part of overseas France enjoying the same administrative status, thus sharing information on both COVID-19 transmission and mortality, and travels monitoring, comparable to mainland France, i.e., “Départements and Régions d’outre-mer” [47,48].

The French departments of French Guiana, Mayotte, Saint Barthélemy, Saint Martin, and Saint Pierre and Miquelon were excluded from this study due to the fact that they are part of regions where the data on COVID-19 and/or travels was either not reported or deemed of little consistent quality [47,48].

French Guiana, Mayotte, and Saint Martin are also featured by significant undocumented, i.e., illegal-cross-border, movements [49,50]. By 3 March 2022, only four cumulative COVID-19 deaths in French Guiana were reported in the time series published by the Géodes platform of the French public health agency—i.e., Santé Publique France—[47], against 393 in the regional epidemiological point published contemporarily by the regional branch of the same organisation [51]. This could reflect a form of complacency from the central agency to bring such regional data out in the open.

Saint Barthélemy and Saint Pierre and Miquelon are too sparsely populated to reach a sufficient sample size for this study.

Thus, only three regions were selected: Guadeloupe and Martinique in the French Caribbean, and Réunion in the Indian Ocean. In combination, these three islands make up two thirds of overseas France’s population, which in turn accounts for 4% of France’s overall population, based on 2003 official estimates [52].

The collection of time series on the notifications of daily new COVID-19 cases and weekly cumulative COVID-19 deaths was completed on 3 March 2022 from Santé Publique France.

Missing case notifications before 13 May 2020 were completed by those reported on WHO’s COVID-19 dashboard. For the record, COVID-19 deaths are notified in France based on electronic death certificates, which in Martinique accounted for 39% of overall death registrations up to March 2020 and 45% up to June 2021, and were more represented by hospitals and care homes than the ambulatory sector [53].

The monthly traffic of air passengers between Paris and Guadeloupe, Martinique, or Réunion was collected from TendanCIEL, the French observatory on commercial air traffic, from January 2020 to January 2022, i.e., the latest data published at the time of collection on 3 March 2022. Values from January to September 2020 were calculated based on the absolute values of year 2021 and the relative values of year 2020 and 2021, compared to 2019.

This focus on the aviation sector is acceptable to the extent that flights from mainland France, i.e., mostly from Paris, are the main gateways to Guadeloupe, Martinique, and Réunion [1]. Flight connections between Paris and overseas France were four times as frequented as international air connections in 2020 [1], ten times as frequent as regional air connections in 2020–2021 [48], and twenty times as frequent as regional maritime services in 2020 in the French Caribbean. In the meantime, the international maritime traffic of passengers was negligible, as cruise ships were being halted since March 2020 [54,55,56].

Unfortunately, TendanCIEL does not distinguish between inbound and outbound traffic. It is conceivable that the monthly data on air traffic are consistent with volume of round trips. In fact, the average length of stay of non-residents was 11 days, by 2020, in Martinique [54], and cannot be deemed longer in Guadeloupe based on hotel reservations [57]. Stays seemed sensibly longer by six days in Réunion, i.e., 17 days, but the last official survey found dates back from before the pandemic, in 2015 [58]. It was unclear from the data, however, whether lengths of stay were equally distributed over months. Summer or winter holidays might be prone to longer stays, but are also made up of longer month periods, i.e., 31-day months (July, August, December, and January). Moreover, other factors (e.g., social unrest, stringent stay-at-home orders, etc.) had the potential to influence stays. Therefore, no correction was applied based on length-of-stay estimates, considering that trends of monthly air traffic was, by and large, sparsely polluted by the outward traffic.

All-time series were converted into day averages.

The value of weekly data on COVID-19 deaths and monthly data on air traffic divided by an interval of days—7, 28, 29, 30, or 31—and computed by week of the year, starting each Monday, or calendar month, starting on the first of each month.

Official indicators from the European Centre for Disease Prevention and Control (ECDC) used to monitor COVID-19 community transmission and mortality in France were computed as follows:

The 14-day moving averages of COVID-19 cases and deaths were calculated between day 13 and day 0.

These were subsequently rated upon 2021 legal population estimates of the French statistics bureau (INSEE), corresponding to 356,029 inhabitants in Martinique, 377,856 in Guadeloupe, 866,181 in Réunion, and 65,447,454 in mainland France [59]. The timeline of public health measures with regard to vaccine and immunity passports, travel quarantines, and states of health emergency were collected from official logbooks of the French central executive [60] or regional representatives in Guadeloupe, Martinique, and Réunion [61,62,63].

### 2.3. Data Analysis

Analyses on COVID-19 outcomes and their time correlations with air traffic were divided in relation to the day the vaccine and immunity passports policy began between mainland France and Guadeloupe, Martinique, or Réunion, i.e., before and since 9 June 2021 [4].

Unpaired t-tests for independent samples were performed using Welch’s correction for unequal variance, as it was meaningful to express COVID-19 deaths by comparing the means, not the medians [64,65,66]. Time correlations were performed using Pearson correlation coefficients compatible with the linear relationships visually observed with scatterplots. Both showed relative robustness to non-normal distributions in large samples, [67,68] as observed while exploring the dataset. Log transformations were applied to COVID-19 cases and deaths while running the analysis on time correlation as a mean of offsetting outlier values. This is a common practice for the analysis of right-skewed time series on COVID-19 [69].

R-4.1.1 software [70] was used to compute the management of data and statistics. The R script and the dataset are provided as Supplementary Materials to this article.

## 3. Results

### 3.1. Description of COVID-19 Transmission and Mortality Compared to the Adoption of Immunity Passports

Daily 14-day notification rates of new COVID-19 (D14NRNC) cases, or COVID-19 community transmission rates, were computed from 16 January 2020, and D14NRNC deaths, or COVID-19 mortality, from 12 January 2020, both until 27 February 2022. Monthly data on air traffic were computed from January 2020 to January 2022.

**Figure 1:**
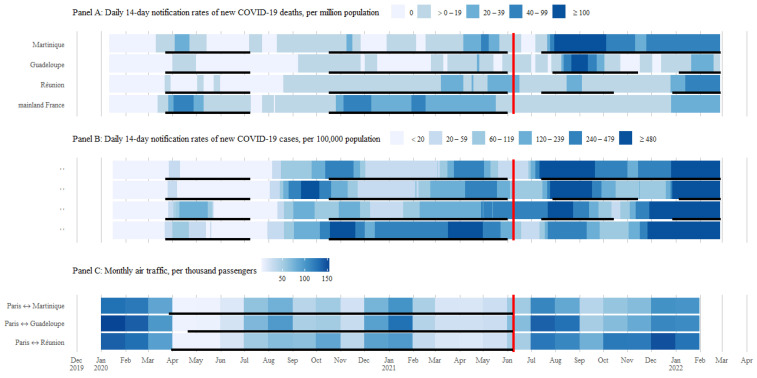
Time series of COVID-19 in overseas and mainland France regarding air traffic with Paris. **Legend:** Crossbars = opening day of COVID-19 vaccine/immunity-based travel bubbles between mainland and overseas France; underlines = states of health emergency (Panels A and B) and mandatory quarantine regimes (Panel C).

Figure 1 shows that COVID-19 mortality in overseas France turned into the highest pandemic records since the adoption of immunity passports, contrary to mainland France (Figure 1). D14NRNC deaths peaked at 511.2 per million population on 8 August 2021 in Martinique, 158.8 on 5 September 2021 in Guadeloupe, and 67.0 on 6 February 2022 in Réunion, while it had peaked at 61.2 on 12 April 2020 in mainland France.

Since the adoption of immunity passports, average D14NRNC deaths were higher in overseas France than in mainland France (115.7 (SD 133.5), 23.5 (SD 38.4), and 21.3 (SD 18.5) for Martinique, Guadeloupe, and Réunion, respectively, versus 11.8 (SD 10.4) for mainland France; three Welch’s *t*-tests *p* < 0.0001). By contrast, D14NRNC deaths were lower in overseas France than in mainland France before the adoption of immunity passports (7.7 (SD 10.4), 2.8 (SD 4.7), and 6.0 (SD 8.2), respectively, versus 20.3 per million population (SD 17.8); all *p* < 0.0001).

D14NRNC cases show similar figures. Rates were lower in Martinique, Guadeloupe, and Réunion than mainland France (89.5 (SD 120.6), 120.0 (SD 153.6), and 100.9 (SD 87.5), respectively, versus 233.0 per 100,000 population (SD 241.1); all *p* < 0.0001) before the adoption of immunity passports. Upon the adoption of immunity passports, these became higher in overseas France than in mainland France, although not statistically significant (1504.7 (SD 1785.8), 1541.0 (SD 2210.3), and 1688.8 (SD 2745.0), respectively, versus 1309.3 (SD 2046.0); *p* = 0.24 for Martinique, *p* = 0.21 for Guadeloupe, and *p* = 0.07 for Réunion).

### 3.2. Timeline of States of Health Emergency in Relation to the Adoption of Immunity Passports

Quarantines were mandatory for all travellers from mainland to overseas France from March/April 2020 to 9 June 2021. These quarantines became waivable by immunity passports from that date onwards. Incidentally, both Caribbean islands had already opened a regional travel bubble system beginning in May 2020. In this intra-Caribbean context, immunity passports were staged from 14 October 2021 to waive mandatory testing for regional travel.

States of health emergency (SoHEs) were enforced three to four times in overseas France versus twice in mainland France (Figure 1). The two first periods of SoHEs were common to the whole of France. In other words, these were blanket for overseas France, insofar as both overlapped the high COVID-19 mortality in mainland France only. Indeed, the second SoHE was discontinued amid a flare of COVID-19 in Réunion, i.e., the 3rd wave. Other than 10 days around the 1 May 2021 in Martinique, neither of these two first SoHEs encompassed a level of COVID-19 transmission or mortality any high ECDC levels of COVID-19 in the French Caribbean. By late summer 2020, COVID-19 cases peaked for the first time in Guadeloupe, though the SoHE remained lifted.

Eventually, a new round of SoHEs was phased-in to overseas France only, five to seven weeks after the adoption of immunity passports. These new overseas-specific SoHEs were differentiated and show timeliness in accordance with the rise of domestic COVID-19 mortality, although unevenly, across the studied French overseas islands.

### 3.3. Time Correlations of COVID-19 and Air Traffic with Paris in Overseas France in Relation to the Adoption of Immunity Passports

**Figure 2:**
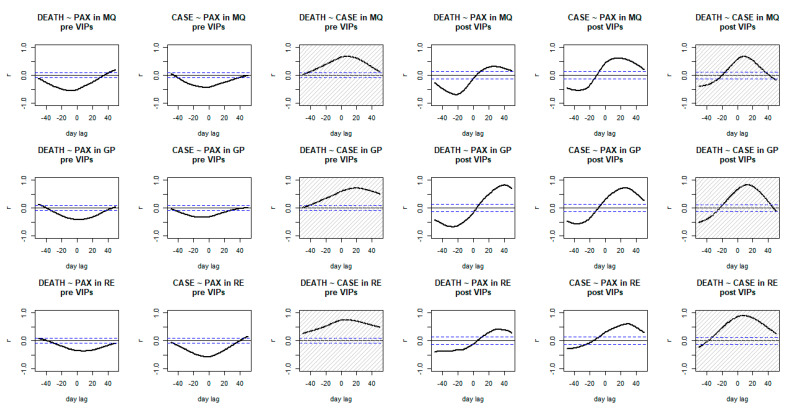
Time-correlations of air traffic with Paris and/or log-scaled 14-day notification rates of new COVID-19 deaths and/or cases in the overseas France. Three columns on the left correspond to the time before introduction of immunity passports. **Abbreviations:** r = Pearson correlation coefficient; GP = Guadeloupe; MQ = Martinique; RE = Réunion; DEATH = 14-day notification rates of new COVID-19 deaths; CASE = 14-day notification rates of new COVID-19 cases; PAX = air traffic of passengers; VIPs = vaccine and immunity passports. **Legend:** dashes = 95% confidence intervals.

According to Figure 2, time correlations between log-scaled COVID-19 transmission and mortality was relatively insensitive to the time of adoption of immunity passports. Before the adoption of immunity passports, log-scaled D14NRNC deaths were maximally correlated to log-scaled D14NRNC cases 8 days before (Pearson coefficient r = 0.67) in Martinique, 20 days before (r = 0.71) in Guadeloupe, and 5 days before (r = 0.74) in Réunion, compared to 9 days before (r = 0.67), 13 days before (r = 0.83), and 8 day before (r = 0.89), respectively, upon the adoption of immunity passports in each island. However, the overall periodicity of time correlation patterns emerged shortened upon the adoption of immunity passports (see Figure 2, grey boxes in columns 3 and 6).

Before immunity passports, the trend of air traffic was concomitantly reversed to the trends of both COVID-19 community transmission and mortality (i.e., the more COVID-19, the less travel took place, and the less COVID-19, the more travel took place). This was featured by air traffic showing moderate or strong negative correlations with log-scaled D14NRNC cases 4 days after (r = −0.43) and log-scaled D14NRNC deaths 7 days after (r = −0.56) in Martinique, compared to 9 days after (r = −0.33) and at day 0 (r = −0.42) in Guadeloupe, and 8 days after (r = −0.37) and at day 0 (r = −0.58) in Réunion.

Upon the adoption of immunity passports, a sizeable leader–follower relationship emerged between air traffic and both log-scaled COVID-19 transmission and mortality. Air traffic suddenly showed sizeable and positive correlations to both log-scaled D14NRNC cases and deaths, 16 days (r = 0.61) and 26 days after (r = 0.31) in Martinique, 26 days (r = 0.72) and 40 days after (r = 0.82) in Guadeloupe, and 29 days (r = 0.60) and 31 days after (r = 0.41) in Réunion.

## 4. Discussion

This study shows that, in distant parts of overseas France, the changes in air traffic started setting pace to a parallel change in the spread of COVID-19 and COVID-19 mortality, when travel bubble systems opened based on vaccine and immunity passports. This is particularly striking, as the change in air traffic and the trends of COVID-19 outcomes seemed to have counterweighted each other beforehand.

Since the adoption of vaccine immunity passports, COVID-19 soared at unprecedented levels across overseas France. Most notably, air traffic emerged as an earlier warning sign of COVID-19 mortality than community transmission in overseas France, and as equally correlated as community transmission to COVID-19 mortality in Guadeloupe.

These findings, based on the crossing of sectoral routine surveillance data, contribute to filling the scientific gap that WHO identified in mid-2021 related to the lack of “real world” empirical evidence to guide public health measures taken in the aviation sector in the context of COVID-19 [25].

### 4.1. Breaking the Circle of Blame

Political elites have a double duty with regards to COVID-19 vaccines and the place of travel and tourism in the recovery from the pandemic. They should ensure that the central and local professionals are informed by evidence not bounded in their own rationality, and that the ordinary population has access to health literacy, rather than being benighted by empty suppositions. COVID-19 measures in the context of travels should be transparent and have attainable objectives for all [71].

However, it would be improper to conclude from this study, as it is the first essay to touch upon the impact of vaccine and immunity passports on COVID-19, that “this is all because of the vaccine or outsiders that import the virus”.

First, what raises an issue in this study is not the COVID-19 vaccine itself, but its reuse for socio-economic policies that are kept unevaluated [24,25,30], and fall outside of health systems’ objectives. Such an immoderate spin is likely to ripple on the peoples’ code of conduct during the pandemic, as it may have compelled travellers to fall into the use of “fake vaccination certificates” [71] or feel a “false sense of security” [12] once they arrived at the destination, especially in the context of mass tourism. While the findings of this study show that COVID-19 vaccine and immunity passports are not good for public health recovery from the pandemic, this should not obscure the fact that the COVID-19 vaccine, if it is used wisely, could spare hospital capacities and save people’s lives [15,72,73].

Second, this study is not set to show the causality or test the hypothesis of COVID-19 importation to overseas France. Indeed, the design of the study used does not allow for this. Testing the hypothesis of an importation of a minimum of COVID-19 cases in overseas France would rely on an analysis of contact-tracing data, which is not currently open [47].

Third, a fine line must be thread not to risk protracting politicisation while accounting for travel interventions in the context of COVID-19 control [71,74], and especially in the context of vaccine hesitancy in overseas France [34]. Notably, many round trips between overseas and mainland France are made by residents for professional or family related reasons. For instance, staying at relatives’ homes and not simply for tourism alone [75]; such a perspective is key to prevent an unfruitful circle of blame between communities, as well as between public institutions and the population at large in a context of COVID-19 recovery attempts.

### 4.2. Methods and Results’ Repeatability

The R script and the dataset of this study are provided in the Supplementary Materials, and feedbacks are welcomed.

By design, this study only included three French overseas territories, but its results could be easily repeated in other territories, providing few preconditions:

First, the surveillance data on COVID-19 time series should be representative of the population, constantly reported over time and with the same methods. It should also be of good quality regarding the post-processing of the data: an erratic correction of aggregated COVID-19 mortality at one given time may reflect “false negative” transmission or mortality, in the worst case.

Rapid changes in the health system hospital capacities, vaccine coverage, or circulation of variants are likely to influence the trend of COVID-19 outcomes, especially mortality. Should such changes occur, it would be important to equate them. For instance, should air evacuations be frequent, the method used to define the place of death registration need to be known. However, this information could not be located based on the official data consulted in this study.

In this study, one key characteristic of the health system is its fragility with regards to COVID-19. Thus, the size effect reported in the findings may well exceed what is observable in different contexts. Such variations in repeated size effects should not only be related to health system inequities, but in the impact of travel and tourism sector relative to the resident population.

Second, the surveillance data on the air traffic should encompass and be representative of the bulk of entries, including, when relevant, air, ground, and maritime border crossings. The issue related to accounting for undocumented travels or illegal migration should be addressed in comparison to official data on traffic. The volume of undocumented travels can be either sizeable or insignificant, and its trend could be parallel, reversed, or simply not correlated to the traffic officially documented during COVID-19 interventions. Notification intervals in time series related to the traffic should be as close as possible, and, if possible, constant over time.

Third, the statistics used to explore the relationship between the time series is based on a linear correlation using the Pearson coefficient. This statistic measures the best fit between the below- and above-average observations of two time series, just as sliding a tracing paper; the more normally distributed the observations and the degree of variation around the average, the more accurate the statistic.

Fourth, choosing a comparable economy (i.e., small island tourism economies–“SITEs”) would make sense to repeat these findings. Among French SITEs, French Polynesia [30] or New Caledonia [76] show strong commonalities with the trends of COVID-19 reported in this study. At the international level, terms of comparison can be found in Barbados [77] or Seychelles [78]. A common feature can also be found in the maritime side, e.g., the clear coincidence in time between the resuming of cruise calls and the advent of COVID-19 mortality in Dominica [5,6,7]. This focus should not imply that vaccine and immunity passports have no impact on COVID-19 outside SITEs, but simply that it is more likely to be noticed in SITEs based on the methods used in this study.

Finally, the advantage of this approach is that it can be easily implemented across different territories, without prohibitive costs—i.e., no added technical, administrative, or ethical challenges -, due to the cross-pollination of sectoral monitoring systems which already exist. Its main disadvantage is that it may overlook the health inequities that are intrinsic in a society. For instance, people employed by the Caribbean travel and tourism sector tend to have a greater buy-in to the COVID-19 vaccine compared to the rest of the population, which is characterised by large rates of unemployment. This is called a “no job, no jab” effect, whereby the people which profit the least from the travel and tourism sector may, paradoxically, bear the maximum cost of the COVID-19 pandemic [79].

### 4.3. The Practicality of the Cross-Sectoral Routine Surveillance

WHO has developed the PROGRESS-CANDALS framework [19], which should serve to analyse health equity and human rights considerations in relation of public health measures taken in the context of COVID-19 and aviation. The PROGRESS acronym stands for “Place of residence; Race or ethnicity; Occupation; Gender and sex; Religion; Education; Socioeconomic status; and Social capital or resources”, and the CANDALS stands for “Citizenship; Ability; Neurotypicality or neurodiversity; Disability; Age; Literacy and/or fluency in a universal language of aviation; and Size, body mass index or body habitus”. Such a focus is clearly essential to inform policymaking and verify whether it is aligned with equal health outcomes for all within a territory. PROGRESS-CANDALS studies should be implemented. However, the confidential nature linked to the data collected requires an active effort of research, which may easily be challenged by a lack of time, resources, political will, or ethical barriers. Time and resources may be lacking in territories that face unprecedented flares of COVID-19 or with less equipped health systems. Ethical barriers may vary depending on territories. Political systems are likely to be structurally biased in SITEs due to competing interests. If worst comes to worst, they may even be dismissive to address the elephant in the room [80], such as the impact of travel and tourism recovery on COVID-19 in SITEs.

Put differently, raising the WHO’s PROGRESS-CANDALS framework should not unduly delay, or divert attention from, the combining of routine surveillance data from the health and transport sectors [81]. Should a pandemic recovery plan go the wrong direction and fail to operate its emergency brake mechanism, the early warning system should send the silent alert; every second counts.

Hopefully, the methods presented in this study can serve as a stepping stone for achievable policy guidance on COVID-19 pandemic preparedness and response in the territories most impacted by the travel sector.

### 4.4. Limits of the Study

This study tries to make the best possible use of the public and routine data made available and is only descriptive, not predictive. No stratification was performed in this study regarding vaccine coverage, the type of variants, populations’ health status or health systems’ performance, as these could not be paired with the time series collected. If these avenues prove to be accessible in the future, it would be useful to explore them in order to refine these findings according to the context.

Overseas France is characterised by limited hospital capacities and a high risk of being overwhelmed [28]. It relied on massive recourse on the French hospital reserve and air evacuations during COVID-19, as was reflected in Martinique since 1 August 2021 [53,82,83,84]. However, the data on hospital reserve and air evacuations cannot be accounted for in the current French open COVID-19 database [47]. This lack of information is, perhaps, to be explained by the large fallback on the health military rescue in the context of the loss of control over COVID-19 [82,83,85]. Moreover, these interventions have certainly helped to curb the outbreaks of COVID-19 in overseas France. Should it flatten the curve of the outbreak, it would mechanically have a bearing on time correlation analyses.

The lack of vaccine coverage of the population and the emergence of new variants, such as Delta, which occurred around the adoption of immunity passports, may well be deemed major and unaddressed confusing factors.

However, death ratios observed in this study before and after the adoption of immunity passports outstrip, by far, international findings comparing Delta and pre-Delta periods among the unvaccinated, when accounting for both the increase in the fatality rate [86] and transmission [87].

Overseas France features persistent and structural health status [88] and health systems deficits compared to mainland France [89]. This context of poor health resources should affect the case fatality rate of COVID-19 [90]. However, it was not possible to locate in the literature why disadvantaged health systems and populations were, paradoxically, better off before the adoption of immunity passports, and why, conversely, the air traffic emerged positively correlated to COVID-19 transmission and mortality afterwards. Under-vaccination and new variants are quite unlikely to explain the changes of patterns in the time correlations observed.

Both air traffic and COVID-19 outbreaks are allegedly influenced by a third factor: the stringency of NPIs. NPIs easing is to travel and tourism what the horse is to the cart, according to UNWTO. The less stringent the NPIs, the more appealing the getaway [22].

NPIs have mushroomed so rapidly that it is difficult to equate them in hindsight [61,62,63]. This can be illustrated by the short-lived enforcement of a testing waiver for vaccine and immunity passports between 14 October 2021 [91] and 27 December 2021 in Guadeloupe [92]. Notably, as in most parts of the world, there is a gap in the literature on the enforcement and adherence to the aviation sector’s NPIs, such as the travel quarantine [25,71], and overseas France is no exception. Thus, this is worth a more detailed analysis in further studies. As an example, overseas France has mostly implemented self-quarantines [4], unlike stringent managed quarantines as in the case of New Zealand [93,94]. The self-quarantine approach only works if travellers show more self-discipline or risk aversion to breaching the law than behaviours to shirk the time and socio-economic losses these may face [95].

This study focuses on COVID-19 transmission and mortality, but this means barely exploring the tip of the iceberg. This study overshadows the wider health impact of air traffic during COVID-19. Health systems in overseas France are, by and large, less equipped than in mainland France [89]. Yet there is currently no detailed time series on all-cause mortality made available for 2021 and 2022 in France, including the French overseas territories [96]. Further studies should account for this missing element upon the release of such time series. Indeed, the French Caribbean, i.e., Guadeloupe and Martinique, and other French overseas territories, such as French Guiana and Mayotte, outdid all French pandemic records of 6-month all-cause excess mortality upon adoption of immunity passports [29].

Finally, it should be noted that the time correlation analyses used were rudimentary. This is excusable, in part, because official data on air traffic lacked fine granularity. More precision than monthly intervals and disaggregation of inward and outward traffic would be useful while disclosing air traffic data. Nevertheless, this study shows sufficient statistical power to meet its primary objectives. However, it has not been possible to retrieve the whole time series of air traffic origins with overseas France outside Paris, due to a lack of data disclosure that was not addressed, despite the COVID-19 pandemic. Paradoxically, despite many efforts, no open time series were located in specialised international agencies such as the International Air Transport Association (IATA) or UNWTO [97] at the time of data collection. This was unexpected, since the latter agency called on the health sector to provide quality evidence on COVID-19 for implementing NPIs. This situation should be addressed inquisitively, as it represents a sizeable gap in policy making in the context of pandemic travel and tourism. As stressed by WHO, “health must remain the key priority” and travel measures in COVID-19 “should be based on risk assessment” [24].

### 4.5. Policy Recommendations

This study is in favour of two complementary approaches: a cross-sectoral routine surveillance, and a disambiguation of health or economic interventions.

First, the more opportunities offered to study the real impact of COVID-19 interventions in the sector of travel and tourism, the better. This implies a whole-of-government sharing of data and risk-assessments to plan and monitor COVID-19 recovery [25,71]. The crossing of trans-sectoral routine surveillance data is quick, affordable, and easy to implement, and should be a strategic priority in COVID-19 recovery plans.

Second, vaccines should be recognised for what they are: instruments of public health. There is no time like the present. Travel-dependent territories should consider building back their economies better, without false expectation on the vaccine. In fact, COVID-19 is spelling the end of the business-as-usual travel and tourism industry, since, unlike the yellow fever, there is so far no COVID-19 vaccine that can prevent transmission, as the International Monetary Fund supports [98]. After climate change, COVID-19 is another tangible crisis that should serve to reform the travel and tourism model [99]. To do so, authorities should rest on the foundations of travel innovations deemed successful in the context of COVID-19. One example is the case of the COVID-19 quarantine period schemes implemented in many parts of the Caribbean before the vaccine and immunity passports era. These quarantine periods were not blanket interventions, but occasions to creatively value social distancing [100]. One of the variations is Dominica’s “safe in the nature” programme, adopted in 2020 [7,101,102], but there are many others.

There is no thankless time and effort to place responsibility on the whole-of-governments and empowering ordinary people for a recovery of health for all from the pandemic [103].

## 5. Conclusions

Since travel bubbles based on vaccine and immunity passports to waive quarantine, air traffic is pacesetting COVID-19 within one month for transmission, and within an additional two weeks for mortality, in overseas France. This case highlights that COVID-19 vaccine and immunity passports can be packaged with a false sense of security. Should countries and economies reopen, authorities need to exercise caution not to forget real world evidence and rely on cross-sectoral routine surveillance. It is essential to inform and disambiguate COVID-19 recovery plans in the travel and tourism sector.

## Supplementary Materials

The following supporting information can be downloaded at: https://www.mdpi.com/article/10.3390/vaccines10060852/s1.
